# Long COVID Syndrome and Cardiovascular Manifestations: A Systematic Review and Meta-Analysis

**DOI:** 10.3390/diagnostics13030491

**Published:** 2023-01-29

**Authors:** Abhigan Babu Shrestha, Aashna Mehta, Pashupati Pokharel, Aakash Mishra, Lukash Adhikari, Sajina Shrestha, Randhir Sagar Yadav, Surakshya Khanal, Ranjit Sah, Behdin Nowrouzi-Kia, Bijaya Kumar Padhi, Vijay Kumar Chattu

**Affiliations:** 1 Department of Internal Medicine, M Abdur Rahim Medical College, Dinajpur 5200, Bangladesh; 2 Faculty of Medicine, University of Debrecen, 4008 Debrecen, Hungary; 3 Maharajgunj Medical Campus, Institute of Medicine, Tribhuvan University, Kathmandu 44613, Nepal; 4 Department of Internal Medicine, Kathmandu Medical College and Teaching Hospital, Kathmandu 21266, Nepal; 5 Department of Internal Medicine, Patan Academy of Health Sciences, Lagankhel, Lalitpur 26500, Nepal; 6 Department of Internal Medicine, KIST Medical College, Patan 14142, Nepal; 7 College of Medicine Jacksonville Program, University of Florida, Gainesville, FL 32611, USA; 8 Harvard Medical School, Boston, MA 02115, USA; 9 Department of Microbiology, Tribhuvan University Teaching Hospital, Institute of Medicine, Kathmandu 44613, Nepal; 10 Dr. D.Y. Patil Medical College, Hospital and Research Centre, Dr. D.Y. Patil Vidyapeeth, Pune 411037, India; 11 ReSTORE Lab, Department of Occupational Science and Occupational Therapy, University of Toronto, Toronto, ON M5S 2E8, Canada; 12 Department of Community Medicine and School of Public Health, Postgraduate Institute of Medical Education & Research (PGIMER), Chandigarh 160012, India; 13 Department of Community Medicine, Faculty of Medicine, Datta Meghe Institute of Medical Sciences, Wardha 442107, India; 14 Center for Transdisciplinary Research, Saveetha Dental College, Saveetha Institute of Medical and Technical Sciences, Saveetha University, Chennai 600077, India

**Keywords:** cardiovascular outcome, COVID-19, long COVID, post-COVID syndrome, dysrhythmias, systematic review and meta-analysis, SARS-CoV-2, tachycardia

## Abstract

(1) Background: Long COVID syndrome is a significant cause of morbidity in COVID-19 patients who remain symptomatic with varied clinical presentations beyond three weeks. Furthermore, the relevance of considering cardiovascular outcomes in post-COVID-19 syndrome is important in the current COVID-19 pandemic; (2) Methods: The Preferred Reporting Items for Systematic Reviews and Meta-Analyses (PRISMA) guidelines were followed for this systematic review and meta-analysis. Systematic searches were conducted from multiple databases without language restrictions until October 8, 2022, to find studies evaluating cardiovascular outcomes such as arrhythmias, myocardium and pericardium diseases, coronary vessel disease, and thromboembolic disorders in post-COVID cases. The pooled odds ratio (OR), and standard mean difference (SMD) with their corresponding 95% confidence intervals (CI) were computed to find the association; (3) Results: Altogether, seven studies with a total of 8,126,462 (cases: 1,321,305; controls: 6,805,157) participants were included in the meta-analysis. Pooled odds ratios of cardiovascular outcomes were significantly higher in post-COVID cases (OR > 1, *p* < 0.05) than in controls. However, the mortality (OR: 4.76, *p* = 0.13), and heart rate variability (SMD: −0.06, *p* = 0.91) between cases and controls were not statistically significant; (4) Conclusions: Significant cardiovascular sequelae in long COVID syndrome highlight the importance of careful cardiac monitoring of COVID-19 patients in the post-COVID phase to address cardiovascular complications as soon as possible; larger-scale prospective studies are required for accurate estimation.

## 1. Introduction

Over the last two years, respiratory disease caused by coronavirus (COVID-19) infection has been a significant cause of morbidity and mortality worldwide. Further contributing to morbidity is the long COVID (post-COVID) syndrome, estimated to affect approximately 43% of confirmed COVID-19 patients who may remain ill beyond three weeks [1]. Post-COVID syndrome is characterized by a wide range of symptoms, including persistent fatigue, sensory deficits, neurocognitive deficits, and cardiovascular conditions such as myocarditis and arrhythmias that last for at least 2 months post-COVID and are unexplained by any other diagnosis [2]. Common cardiovascular symptoms in the post-COVID phase are chest pain, palpitation, shortness of breath with exertion, pre-syncope, fatigue, and pedal edema. However, the pathophysiology behind the cardiovascular manifestations of COVID syndrome is unclear.

Oxidative stress from a cytokine storm during active infection can affect the mitochondria of myocardial cells [1]. In addition to parasympathetic and sympathetic dysfunction caused by oxidative stress, this can result in changes in heart rate and blood pressure [1,3]. Approximately 5% of patients experience chest pain, and palpitations were reported in 9% of patients 6 months after follow-up, which can be attributed to autonomic dysfunction [1]. However, there is a conflicting body of evidence on whether parasympathetic or sympathetic dysfunction dominates the clinical picture in patients with long COVID experiencing chest pain and/or palpitations [3].

Myocardial injury can lead to myocardial infarction and heart failure, which occur acutely, while myocarditis and pericarditis may develop chronically. One small study performed on healthy college athletes with mild COVID-19 demonstrated that 15% of the study participants had evidence of myocarditis on magnetic resonance imaging [4]. Recent research complemented by echocardiographic findings has determined the incidence of cardiovascular complications of COVID-19, such as right ventricle failure at 26.3%, left ventricle failure at 18.4%, diastolic failure at 13.2%, and pericardial perfusion at 7.2% [1]. However, it is unclear whether these cardiovascular conditions persist in long COVID patients or reverse post-infection. Another aspect to consider is detecting preclinical myocardial injury. The reliability of determining structural abnormalities on echo and laboratory abnormalities such as troponin levels to reflect the persistence of cardiovascular disease warrants further investigation. Furthermore, the relevance of considering cardiovascular outcomes in the post-COVID-19 syndrome in patients with preexisting cardiovascular disease has become significant in the provision of quality cardiac care. 

As a result, the objective of this systematic review and meta-analysis is to conduct comprehensive evaluations of cardiovascular outcomes in post-COVID syndrome, so as to guide clinical practice for the early detection and management of post-COVID cardiovascular sequelae. 

## 2. Materials and Methods

This study was piloted with Preferred Reporting Items for Systematic Reviews and Meta-analyses (PRISMA) guidelines [5]. The PRISMA checklist is presented in the Supplementary File (Supplementary S1). Additionally, the study has been registered in PROSPERO with ID CRD42022366930 (link: https://www.crd.york.ac.uk/prospero/display_record.php?ID=CRD42022366930, accessed on 23 November 2022).

### 2.1. Search Strategy

Three reviewers (ABS, AM, and LA) performed a rigorous literature search on multiple databases, including PubMed, Cochrane Library, Google Scholar, and Science Direct, from inception until 8 October 2022, using relevant medical subject headings (MeSH) terms and keywords: post-covid, long-covid, long-haul covid, and cardiovascular outcomes. Search terms were further connected with suitable Boolean operators “or” and “and”. The detail of the search strategy is shown in the Supplementary File (Supplementary S2).

### 2.2. Selection Criteria

#### 2.2.1. Inclusion Criteria

We included studies with patients aged ≥ 18 years and with cases and controls; where a case was defined as COVID-19 positive at least 4 weeks ago and tested by recombinant polymerase chain reaction (RT-PCR) and/or high-resolution computed tomography (HR-CT) scan and controls from healthy people (no COVID-19 infection), and assessing cardiovascular outcomes like dysrhythmias (atrial fibrillation, sinus tachycardia, ventricular arrhythmias, atrial flutter, and cardiac arrest), inflammatory disease of the heart or pericardium (pericarditis, and myocarditis), ischemic heart disease (acute coronary disease, myocardial infarction, ischemic cardiomyopathy, and angina), thromboembolic disorders (pulmonary embolism, deep vein thrombosis, transient ischemic attacks, and stroke), also including major adverse cardiovascular event (MACE): which is a composite of myocardial infarction, stroke, and all-cause mortality, and any cardiovascular outcome and mortality due to any cause. We did not impose any time or language constraints.

#### 2.2.2. Exclusion Criteria

Case reports, case series, review articles, editorials, commentary, poster presentations, letters to editors, conference presentations, correspondence, and studies with insufficient data were excluded. Furthermore, studies where a single arm was presented without comparators and with non-compliant outcomes were excluded from our study. 

### 2.3. Study Selection

The four databases were searched systematically by three authors (A.B.S., A.M., and L.A.). The retrieved studies from the electronic databases were imported into Rayyan software, where the duplicate articles were debarred electronically and then manually. Based on the eligibility criteria, three authors independently carried out abstract title screening on all selected studies, and then the full texts of the selected articles were reviewed. Additionally, we searched the reference list of relevant articles to identify any potential papers. Disagreements between reviewers were resolved by the author (P.P.).

### 2.4. Data Extraction

Three authors separately extracted the data from the included studies in a compatible format, considering key characteristics including author, publication year, type of study, sample size (cases and controls), comorbidities, hospital visits, mortality, and final clinical outcomes of both groups. The data were extracted into a shared Microsoft Excel spreadsheet.

### 2.5. Quality Assessment

To evaluate the quality of the included studies, we used the Newcastle-Ottawa Scale for non-randomized studies [6]. Using the tool as a checklist, the quality of each of the included studies was evaluated independently by two authors (PP and ABS). The mean score of two authors was taken for the final decision, and articles with a mean score ≥5 out of 10 (across the three parts) were included in the analysis.

### 2.6. Statistical Analysis

Baseline continuous variables were represented as mean (±standard deviation); however, dichotomous variables were summarized as percentages. The study pooling was performed using the DerSimonian and Laird random effects model for the study variation [7]. Outcomes were pooled as odds ratios (OR) and standard mean differences (SMD) for continuous variables with their corresponding 95% confidence intervals (95% CI), respectively. Statistical significance was considered if the two-tailed *p*-value was less than 0.05. The heterogeneity among the included studies was assessed using the Higgins I-squared (I^2^) model, with I^2^ values < 75% as mild-moderate and ≥75% considered high. Additionally, meta-regression analysis was conducted to find the source of heterogeneity. All statistical analysis, including graphical illustrations, was done using STATA (version 17.0, StataCorp, College Station, TX, USA). 

## 3. Results

### 3.1. Study Selection

**Figure 1:**
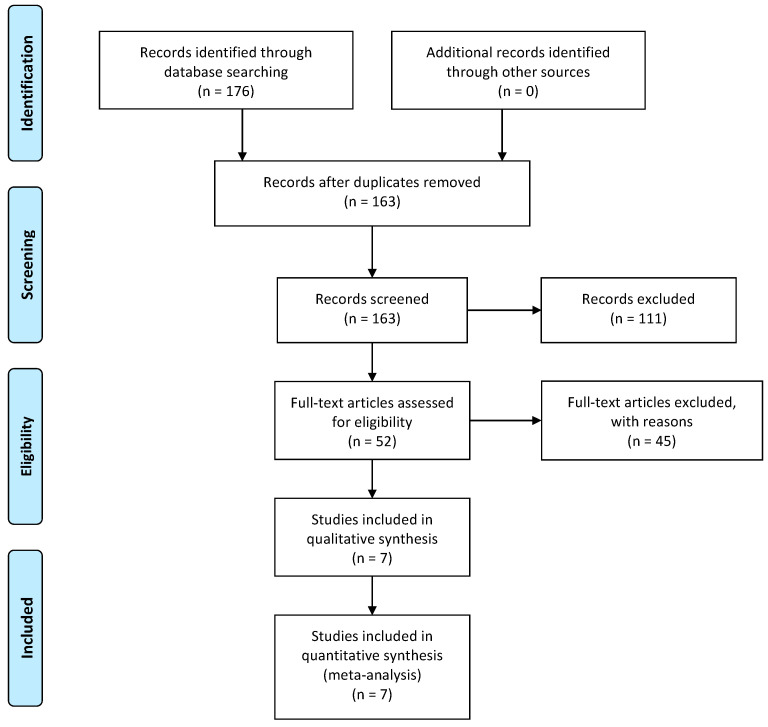
PRISMA diagram showing the study selection process.

Through a comprehensive preliminary database search, we identified 176 relevant studies, including 76 from PubMed, 82 from Google Scholar, 8 from the Cochrane Library, and 10 from Science Direct. Duplicates were screened and excluded after importing articles to Rayyan and confirmed manually. Altogether, 13 duplicate articles were removed. Screening of the titles and abstracts further excluded 52 studies based on our inclusion and exclusion criteria. Full-text articles were analyzed when needed to remove a study lacking sufficient and/or irrelevant data. Finally, a total of 7 studies were included in the meta-analysis. The PRISMA flow diagram for the study selection process is shown below (Figure 1).

### 3.2. Patient and Study Characteristics

Out of the seven studies, three were retrospective cohort studies [8,9,10], one was a prospective cohort study [11], two were prospective observational studies [12,13], and one was a retrospective observational study [14]. Altogether, 8,126,462 participants (case: 1,321,305; control: 6,805,157) were included in the meta-analysis. All studies were published between December 2021 and August 2022. There was a worldwide distribution of studies: two from the United States, one from Turkey, one from India, two from England, and one from Israel. The total number of males in the case was 651,905; in control, it was 5,606,196. The entire population was 18–80 years old, with a male predominance. Only two studies [12,13] included healthy adults with no comorbidities. All studies adopted a duration of 4 weeks following infection with COVID-19 to evaluate post-COVID outcomes. The baseline demographics of the study participants are listed in Table 1.

Xie et al. [10] longitudinally followed cohorts to evaluate the risk and 12-month burden of prespecified incident cardiovascular outcomes compared to the contemporary control group and, separately, to the historical control group. Asarcikli et al. [12] included patients between 12 and 26 weeks after COVID-19 infection, using a non-COVID-era database as the control group. Shah et al. [13] prospectively evaluated patients within 30 to 45 days of infection and enrolled healthy volunteers as controls. Rezel-Potts et al. [11] evaluated longitudinal electronic health records from primary care and compared COVID-19 patients with matched population controls from one year before a COVID-19 diagnosis to one year after. Szekely et al. [14] prospectively evaluated patients and compared them with historical control subjects. Ayoubkhani et al. [8] conducted an observational, retrospective, matched cohort study of individuals with candidate individuals from the general population as controls. Wang et al. [9] conducted a retrospective cohort study and followed up with patients after 30 days of COVID-19 infection. The control group included individuals who were negative for COVID-19 and did not show any symptoms of COVID-19.

### 3.3. Risk of Bias Assessment

The risk of bias in all included studies was assessed using the Newcastle-Ottawa scale. Studies with a mean score greater than or equal to 7 were considered “low risk”, while studies with a mean score less than 7 were considered “high risk”. All seven studies had a mean score of ≥7 and were considered “low risk”. So, all studies were included in the final analysis. The details of the quality assessment are illustrated in the Supplementary File (Supplementary S3).

### 3.4. Meta-Analysis

#### 3.4.1. Electrophysiological Abnormalities (Arrhythmias)

The odds of having electrophysical abnormalities in cases compared to controls by pooling the results from two studies were: atrial arrhythmia [OR: 2.05, (95% CI: 1.24–2.85), I^2^ = 86.48%, *p* < 0.001], sinus bradycardia [OR: 1.57, (95% CI: 1.50–1.63), I^2^ = 25.51%, *p* < 0.001], sinus tachycardia [OR: 1.75,(95% CI: 1.60–1.91), I^2^ = 85.12%, *p* < 0.001], supraventricular tachycardia [OR: 1.77, (95% CI: 1.42–2.12), I^2^ = 89.79%, *p* < 0.001], ventricular arrhythmia [OR: 1.71, (95% CI: 1.48–1.95), I^2^ = 90.36%, *p* < 0.001], and cardiac arrest [OR: 2.08, (95% CI: 1.40–2.76), I^2^ = 88.17%, *p* < 0.001]. Additionally, pooling the results from three studies, the heart rate variability between the cases (treated group) and the controls was [SMD: −0.06, (95% CI: −1.03–0.92), I^2^ = 95.25%, *p* = 0.91].

#### 3.4.2. Cardiac Tissue Abnormalities

Similarly, by pooling the results from two studies, the odds of having cardiac tissue abnormalities in cases compared to controls were: pericarditis [OR: 1.72, (95% CI: 1.49–1.94), I^2^ = 50.54%, *p* < 0.001], myocarditis [OR: 4.90, (95% CI: 3.55–6.24), I^2^ = 0%, *p* < 0.001], ischemic cardiomyopathy [OR: 2.28, (95% CI: 1.24–3.32), I^2^ = 94.31%, *p* < 0.001], and non-ischemic cardiomyopathy [OR: 2.02, (95% CI: 1.24–2.79), I^2^ = 98.87%, *p* < 0.001].

#### 3.4.3. Coronary Vessel Abnormalities

From the pooling of two studies, the odds of having coronary vessel disease in cases compared to controls were angina [OR: 1.60, (95% CI: 1.42–1.78), I^2^ = 69.48%, *p* < 0.001], acute coronary disease: [OR: 1.85, (95% CI: 1.54–2.17), I^2^ = 88.17%, *p* < 0.001], and myocardial infarction [OR: 1.60, (95% CI: 1.42–1.78), I^2^ = 69.48%, *p* < 0.001].

#### 3.4.4. Thromboembolic Disorders

Moreover, pooling the results from three studies, the odds ratio of thromboembolic abnormalities in cases compared to controls were: pulmonary embolism [OR: 2.76, (95% CI: 2.50–3.02), I^2^ = 53.10%, *p* < 0.001], stroke [OR: 1.39, (95% CI: 1.15–1.63), I^2^ = 91.90%, *p* < 0.001]. Also, pooling the two studies, we obtained the odds ratios for deep vein thrombosis [OR: 1.98, (95% CI: 1.77–2.19), I^2^ = 76.50%, *p* < 0.001], transient ischemic attack [OR: 1.49, (95% CI: 1.39–1.59), I^2^ = 0%, *p* < 0.001], and total embolic disorder [OR: 2.22, (95% CI: 1.73–2.72), I^2^ = 58.38%, *p* < 0.001], in the cases compared to the controls.

#### 3.4.5. MACE and Mortality

Upon pooling the results from three studies, the odds of having MACE in cases compared to controls were [OR: 2.11, (95% CI: 1.71–2.50), I^2^ = 98.85%, *p* < 0.001]. The odds for cardiogenic shock obtained by pooling two studies were [OR: 2.14, (95% CI: 1.73–2.55), I^2^ = 18.26%, *p* < 0.001]. Similarly, the mortality odds obtained by pooling two studies were higher in cases [OR: 4.76, (95% CI: 1.44–10.96), I^2^ = 99.77, *p* = 0.13].

### 3.5. Meta-Regression

To find the source of heterogeneity, meta-regression analysis was done for individual cardiovascular outcomes. From regression analysis, the causes of heterogeneity were the patients’ age (*p* < 0.05), male sex (*p* < 0.05), and comorbidities (*p* < 0.05). The individual *p*-values corresponding to the source of heterogeneity are presented in the Supplementary File (Supplementary S5). However, fewer studies (n = 7) have given less power to the regression analysis.

## 4. Discussion

In this meta-analysis, data from a total of 8,126,462 individuals (cases: 1,321,305; controls: 6,805,157) was extracted from 7 published studies to find the association between post-COVID syndrome and cardiovascular abnormalities. We found that post-COVID cases, compared to healthy controls, had higher odds of cardiovascular problems such as electrophysiological abnormalities, coronary vessel diseases, thromboembolic disorders, and diseases of cardiac tissue such as pericarditis and myocarditis.

Globally, as of 17 November 2022, there have been 633,263,617 confirmed cases of COVID-19, including 6,594,491 deaths [15]. Yet, the exact pathomechanism underlying the association between COVID-19 infection and the development of cardiovascular complications in the post-acute phase of the infection remains unclear. Mechanisms such as persisting damage post-viral invasion of cardiac myocytes and consequent cellular necrosis, infection, and inflammation affecting endothelial cells, as well as viral transcription alteration of various cell lines in cardiac tissue, can promote the development of myocarditis [16]. Other effects in the inflammatory cascade include complement pathway activation and cytokine-mediated coagulopathy, microangiopathy, and downregulation of angiotensin-converting enzyme-2 (ACE-2) leading to dysregulation of the renin-angiotensin-aldosterone system (RAAS), which increase the propensity towards thromboembolism [17,18]. Another underlying mechanism, including the incorporation of the SARS-CoV-2 genome in the DNA of infected human cells, has also been implicated in the continued activation of the immune and inflammation-mediated coagulation cascades. Autonomic dysfunction prevalent during and post-infection may provide a possible explanation for arrhythmias [19].

**Figure 2:**
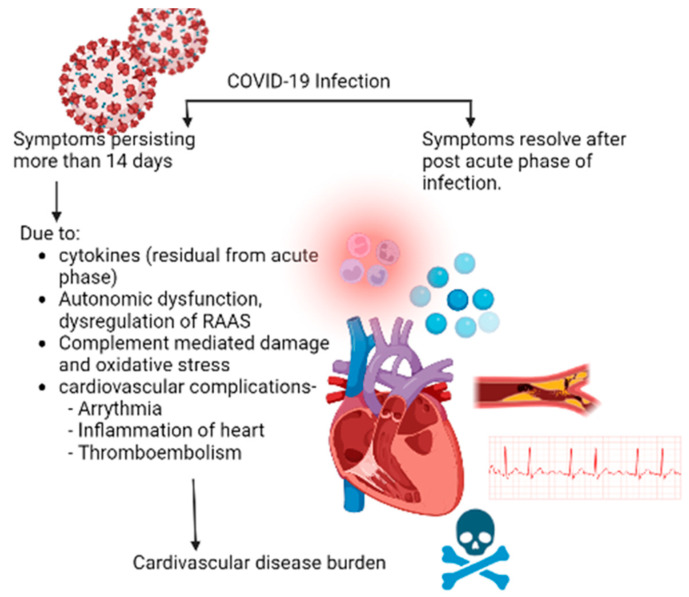
Flow diagram for post-COVID cardiovascular manifestations.

Additionally, persistent elevation of pro-inflammatory cytokines and activation of multiple growth factor signaling pathways could induce fibrosis and scarring of cardiac tissue [20,21]. These pathways provide a plausible explanation for the vast range of cardiovascular complications in long covid syndrome, as evidenced by this analysis. Figure 2 shows the flowchart for cardiovascular manifestations in post-COVID syndrome.

Furthermore, post-COVID cardiovascular manifestations are the consequences of altered physiology of the cardiovascular system and mechanical damage to the cardiac system during the acute phase of COVID-19 infection. Some key mechanisms for physiological alterations in COVID-19 are post-inflammatory residual damage, immune system dysregulation by cytokines, complications after critical illness, an inadequate antibody response, and an ongoing underlying viral infection [22].

In our meta-analysis, the odds of developing myocarditis were around 5-fold, pericarditis was 1.5-fold, and cardiomyopathy was 2-fold in the post-COVID-19 phase. The COVID-19 infection is associated with an intense host inflammatory response and an excessive release of cytokines, leading to a cytokine storm. Interleukin-6 (IL-6), a major driver of cytokine storms, promotes vascular leakage and impairs myocardial contractility, including papillary muscle [23,24]. Direct invasion of cardiac myocytes by SARS CoV-2 is also responsible for myocarditis [25]. Furthermore, sympathetic surge, microcirculatory dysfunction, a proinflammatory state, and vasospasm have been proposed to cause cardiomyopathy in COVID-19 patients [26].

The odds of developing coronary vessel diseases such as angina (OR 1.6) and myocardial infarction (OR 1.6) were also significantly higher in long-COVID cases. Ischemic myocardial damage in COVID-19 is because of the systemic inflammatory response causing plaque rupture in coronary vessels [27,28]. Furthermore, in severe COVID-19 infection, the imbalance between increased myocardial oxygen demand due to cytokine storm and decreased oxygen supply to cardiomyocytes as a result of acute respiratory distress syndrome causes varying degrees of myocardial injury [29]. 

Similarly, the odds of developing thromboembolic changes such as deep vein thrombosis (OR 1.98), pulmonary embolism (OR 2.76), TIA (1.49), and stroke (OR 1.39) are also higher in post-COVID syndrome. The endothelial infection in SARS-CoV-2 infection leads to inflammation of blood vessel walls and, consequently, the death of the endothelial cell [30], which ultimately promotes vasoconstriction, turbulence in blood flow, thrombus, and atheroma formation [31]. COVID causes ACE-2 expression to be downregulated, resulting in low angiotensin I-7 levels. This further results in a pro-inflammatory and pro-thrombotic effect of angiotensin II [32]. Furthermore, prolonged immobilization, complement activation, and high levels of anti-phospholipid antibodies are other factors with pro-thrombotic properties that are prevalent among patients with severe COVID-19 infection [33,34]. All these factors favor Virchow’s triad and ultimately predispose to arterial and venous thrombosis. Venous thrombosis manifests as DVT and PE, whereas arterial thrombosis manifests as TIA, stroke, or myocardial infarction.

Additionally, the odds of developing atrial and ventricular arrhythmias, including cardiac arrest, are higher in long-covid cases. The probable causes of electrophysiological disturbances are autonomic disturbances, systemic inflammation and cytokine release [35], hypoxia and myocardial injury [36], and electrolyte and volume imbalance [37]. The odds of having myocardial injury manifest as angina and myocardial infarction are higher in the post-covid phase, thus increasing the odds of arrhythmias. However, the heart rate variability between the cases and controls was not statistically significant (*p* = 0.91). This is because both tachyarrhythmias and bradyarrhythmias were prevalent in the cases.

The analysis highlights the obvious heterogeneity in the results due to age, gender, and the presence of comorbidities. Despite the possible heterogeneity, there is an increased incidence of cardiovascular complications in COVID syndrome. Other studies included in this analysis also report an increased 12-month cardiovascular disease morbidity regardless of age, gender, and comorbidities such as chronic kidney disease, diabetes mellitus, and hypertension, thereby establishing the role of COVID-19 in the pathomechanism of cardiovascular complications. The severity of symptoms and the severity of clinical presentation in the post- COVID phase may also be influenced by the severity of the acute phase of COVID-19 infection, as well as hospitalization status and care quality during the acute phase. 

This meta-analysis is the first to delineate the association of various cardiovascular outcomes with post-COVID-19 syndrome and has several strengths. In patients with a long COVID syndrome, this study discovered a link between the cardiovascular outcomes except heart rate variability. This can be because both tachycardia and bradycardia have been observed in long covid syndrome. With over 633 million people worldwide affected by COVID-19, the findings reported in this study have broad utility and are critical to infectious disease and cardiovascular patient outcomes. The results of this analysis have included a large sample size representative of different countries, different age groups, and gender. Moreover, this reporting indicates the relevance of continued research and reporting on cardiovascular outcomes and long COVID syndrome to better anticipate and identify high-risk patient groups that can be monitored and managed early for the development of any cardiovascular events. 

Despite significant utility, this review has some limitations to address. Although clearly defined, the selection criteria and analysis did not stratify for gender, and therefore, gender-related differences in cardiovascular pathology were not included in this systematic review. The exact timeline of the development of various cardiovascular sequelae and the presence of preexisting cardiovascular disease or risk factors were not stratified, which could potentially influence the incidence of cardiovascular events in long COVID syndrome. Gender and preexisting cardiovascular disease are other factors that require stratification in future studies to make more comprehensive conclusions on the development of cardiovascular disease in long COVID syndrome and subsequent mortality. A relative scarcity of randomized studies was noticed during the review, which is required to further verify our findings. Furthermore, the included studies did not report baseline odds for the controls, so the comparison made based on the pooled odds ratio might provide an incomplete description of the data. Therefore, the results of this meta-analysis should be interpreted carefully.

## 5. Conclusions

With ever-increasing COVID-19 cases, evidence of the association between COVID-19 pathology and cardiovascular manifestations is paramount. Considering this, our meta-analysis delineates that long COVID syndrome is associated with an increased burden of cardiovascular manifestations. Further this study emphasizes to conduct large-scale, multicenter researches to address the heterogeneity of post-COVID cardiovascular sequelae in different age groups and genders, as well as a pinpointing of the exact pathomechanism that can provide valuable evidence and aid in the identification of novel therapeutic targets to prevent the development of cardiovascular disease in COVID-19 patients.

## Figures and Tables

**Table 1:** Baseline study characteristics.

Study	Country	Study Design	Number of Participants (Cases)	Male (Percentage)	Age in Years; Median/Mean; Range	Evaluation Following Infection	Comorbidities Assessment
Ayoubkhani et al., 2021 [8]	England	Retrospective cohort study	47,780	26245 (54.9)	Cases:64.5 ± 19.2 Controls: NA	Mean follow-up of 140 days, SD 50 days	Yes
Wang et al., 2022 [9]	United States	Retrospective cohort study	690,892	298223 (43.2)	Cases: 43.2 ± 16.2 Controls: 44.5 ± 17.0	After 30 days	Yes
Xie et al., 2022 [10]	United States	Retrospective cohort study	153,760	NA	Cases: 61.42 ± 15.64 Controls: 63.46 ± 16.23	After 30 days	NA
Rezel-Potts et al., 2021 [11]	England	Prospective matched-cohort study	428,650	167,867 (45)	Cases/Controls: 35 (22 to 50)	Post-acute: 5 to 12; Long: 13 to 52 weeks from index	NA
Asarcikli et al., 2022 [12]	Turkey	Retrospective observational study	60	23 (38.3)	Cases: 30 (26–42) Controls: 39 (31–49)	After 12 weeks	None; healthy individuals
Shah et al., 2022 [13]	India	Prospective observational study	92	54 (58.7)	Cases: 50.6 ± 12.1 Controls: 51.8 ± 4.2	After 30 days	None; healthy individuals
Szekely et al., 2021 [14]	Israel	Prospective observational study	71	47 (66)	Cases: 52.6 ± 16 Controls: 53.6 ± 16	After 3 months	Yes

## Data Availability

The data presented in the study are available on request from the corresponding authors.

## Supplementary Materials

The following supporting information can be downloaded at: https://www.mdpi.com/article/10.3390/diagnostics13030491/s1, Supplementary S1: PRISMA Checklist; Supplementary S2: Search Strategy; Supplementary S3: Quality Assessment (New Castle Ottawa Scale for Non-randomized Studies); Supplementary S4: Forest Plots; and Supplementary S5: Meta-regression Analysis.
